# Accuracy of Perceived Estimated Travel Time by EMS to a Trauma Center in San Bernardino County, California

**DOI:** 10.5811/westjem.2016.5.29809

**Published:** 2016-06-21

**Authors:** Michael M. Neeki, Colin MacNeil, Jake Toy, Fanglong Dong, Richard Vara, Joe Powell, Troy Pennington, Eugene Kwong

**Affiliations:** * Arrowhead Regional Medical Center, Department of Emergency Medicine, Colton, California; † Western University of Health Sciences, College of Osteopathic Medicine of the Pacific, Pomona, California; ‡ City of Rialto Fire Department, Rialto, California

## Abstract

**Introduction:**

Mobilization of trauma resources has the potential to cause ripple effects throughout hospital operations. One major factor affecting efficient utilization of trauma resources is a discrepancy between the prehospital estimated time of arrival (ETA) as communicated by emergency medical services (EMS) personnel and their actual time of arrival (TOA). The current study aimed to assess the accuracy of the perceived prehospital estimated arrival time by EMS personnel in comparison to their actual arrival time at a Level II trauma center in San Bernardino County, California.

**Methods:**

This retrospective study included traumas classified as alerts or activations that were transported to Arrowhead Regional Medical Center in 2013. We obtained estimated arrival time and actual arrival time for each transport from the Surgery Department Trauma Registry. The difference between the median of ETA and actual TOA by EMS crews to the trauma center was calculated for these transports. Additional variables assessed included time of day and month during which the transport took place.

**Results:**

A total of 2,454 patients classified as traumas were identified in the Surgery Department Trauma Registry. After exclusion of trauma consults, walk-ins, handoffs between agencies, downgraded traumas, traumas missing information, and traumas transported by agencies other than American Medical Response, Ontario Fire, Rialto Fire or San Bernardino County Fire, we included a final sample size of 555 alert and activation classified traumas in the final analysis. When combining all transports by the included EMS agencies, the median of the ETA was 10 minutes and the median of the actual TOA was 22 minutes (median of difference=9 minutes, p<0.0001). Furthermore, when comparing the difference between trauma alerts and activations, trauma activations demonstrated an equal or larger difference in the median of the estimated and actual time of arrival (p<0.0001). We also found month and time of day to be associated with variability in the difference between the median of the estimated and actual arrival time (p=0.0082 and p=0.0005 for month and time of the day, respectively).

**Conclusion:**

EMS personnel underestimate their travel time by a median of nine minutes, which may cause the trauma team to abandon other important activities in order to respond to the emergency department prematurely. The discrepancy between ETA and TOA is unpredictable, varying by month and time of day. As such, a better method of estimating patient arrival time is needed.

## INTRODUCTION

Trauma is the leading cause of death among Americans between the ages of 1 to 46 in the United States.^1^,^2^ Trauma patients represent a heterogeneous group that are affected by a myriad of injury mechanisms. These patients often require rapid physician evaluation followed by a multitude of diagnostic procedures, imaging studies and therapeutic treatments.^3^ As such, trauma places a significant socioeconomic burden on the U.S. healthcare system and society as a whole. The Centers for Disease Control and Prevention estimates the cost of trauma to be $406 billion per year, a figure that encompasses both lost productivity and healthcare costs.^1^

**Figure 1:**
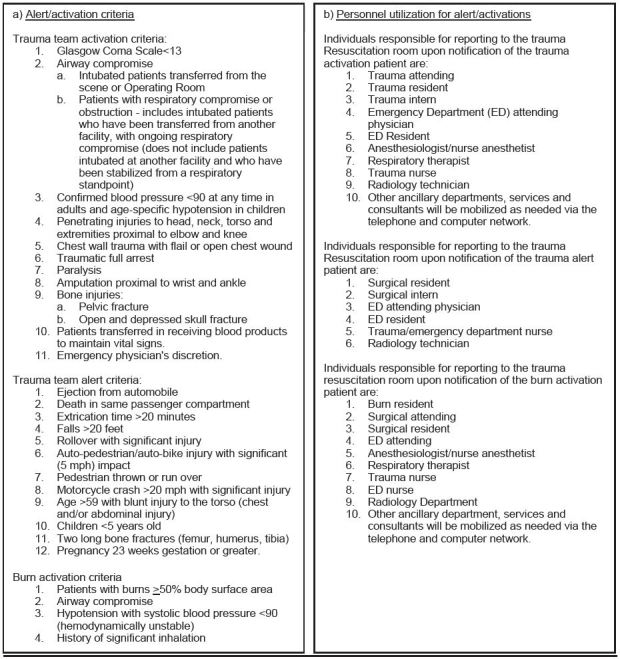
Trauma alert and activation criteria and the corresponding personnel utilization.

Following the introduction of Advanced Trauma Life Support in the 1970s, a coherent response to trauma has been shown to reduce mortality in this patient group.^4^–^9^ Patients with multisystem injury are assessed by an organized team of professionals from a variety of specialized services.^8^,^10^ This multidisciplinary group is known as the trauma team (Figure 1).

When a patient meets a pre-defined criterion, trauma systems are activated which includes trauma team notification (Figure 1). Altered resource allocation as a result of trauma system activation has the potential to create ripple effects throughout hospital operations. Previous studies indicate that trauma team activation significantly delayed initial physician examination of other emergency department (ED) patients and often increased ED length of stay.^11^ Imaging resources, operating rooms and laboratory services may also be placed on hold for a trauma patient’s potential need, which further contributed to delays in the care of patients. As such, the timing of trauma system activation is of critical importance.

At present, many trauma centers rely solely on a prehospital provider’s estimation of travel time relayed over radio or telephone in order to determine the timing of trauma system activation. Emergency medical services (EMS) personnel often give this estimation while en route to the receiving trauma center. However, unforeseen factors may affect patient transport time such as traffic or weather fluctuations, reducing the likelihood that a patient will arrive at the estimated arrival time. Additionally, a past study reported that paramedic’s ability to accurately predict transportation time within two minutes of the actual duration was only 47% of the time.^12^

Accurately predicting patient arrival time has the potential to benefit not only the trauma patient, but also other hospitalized patients at the receiving trauma center. While a patient arriving early may result in lack of ED preparedness or incomplete trauma team assembly, a patient arriving late has the potential to expend valuable hospital resources and inappropriately divert care away from other patients. The current study aimed to assess the accuracy of prehospital estimated arrival time in comparison to the actual arrival time of EMS crews at a high acuity Level II trauma center in Colton, California. We hypothesized that EMS personnel often underestimate patient transport time leading to a discrepancy in estimated time of arrival (ETA) in comparison to actual time of arrival (TOA). Through a greater understanding of this time discrepancy, strategies can be developed to improve the flow of ED patient care, with a future goal of reducing length of stay and improving overall patient outcomes.

## METHODS

### Study Setting

This study took place at Arrowhead Regional Medical Center (ARMC) located in Colton, CA. ARMC is a 456-bed acute care teaching facility and a Level II trauma center that uses a two-tiered trauma activation system. ARMC is the only American College of Surgeons-verified Level II trauma center serving San Bernardino County, CA.^13^ The ED at ARMC is the second busiest in the state of California with more than 116,000 annual visits.^13^ Additionally, more than 12 ground and air providers transport patients to ARMC. These licensed providers, including paramedics and emergency medical technicians (EMT), operate within the 20,000 square miles of San Bernardino County and provide coverage for a mix of urban and rural communities with a total population of over 21 million people.^14^,^15^

While at the scene or en route with a trauma patient, EMS personnel contact the Mobile Intensive Care Nurse (MICN) at ARMC and the trauma is categorized as an alert or activation based on classification criteria (Figure 1). If the MICN determines that a patient meets the alert or activation criteria, he or she activates trauma systems 15 minutes prior to patient arrival when possible, including notification of the multidisciplinary trauma team via the trauma pager system (Figure 1). At the first contact with EMS, a time of initial contact, ETA and name of the transporting provider are recorded by the MICN. Upon patient arrival at the trauma bay, an actual arrival time is recorded. Paramedics and EMTs also record a time of initial contact and actual arrival time. These data are subsequently entered into the Surgery Department Trauma Registry.

### Patients

We conducted a retrospective review to identify trauma patients transported to ARMC between January 1, 2013, and December 31, 2013. All alert and activation classified traumas that contained the time of initial contact by EMS personnel, ETA, TOA and transporting EMS provider were included in the current study. We excluded those with missing time information. Additionally, traumas with an arrival time noted as earlier then the call time, indicating that the patient had arrived to ARMC without prior notification, were excluded. This study was approved by the ARMC Institutional Review Board.

In considering all alert and activation classified traumas, we included transports by only four agencies – American Medical Response (AMR), Ontario Fire, Rialto Fire and San Bernardino County Fire. These four agencies were chosen based on the volume of patients that they transported to ARMC, coverage area and ability to transport directly to ARMC. These four agencies comprised 86% of includible trauma alerts and activations. Additionally, these agencies transported directly from the scene to ARMC without handoffs. In comparison, other local ground providers based farther from ARMC must transfer their patient to another ground or air provider if they wish to send to ARMC. We excluded these transports to ensure consistency in the conditions under which EMS personnel made travel time estimations.

For data analysis purposes, if EMS personnel gave ETA as a time interval, the midpoint was used to calculate median ETA. We combined data points for all subdivisions of San Bernardino County Fire for calculations.

### Statistical analysis

The primary outcome was the difference between the median of ETA and TOA. Additional variables assessed include time of day and month during which the transport took place. We analyzed data using the SAS software for Windows version 9.3 (Cary, NC). Descriptive statistics were presented as median and interquartile for continuous variable, and frequencies and proportions for categorical variables. We conducted non-parametric Wilcoxon Rank Sum-test to compare whether or not the difference of median ETA and TOA was different from zero. Kruskal-Wallis rank test was conducted to identify whether the difference of median ETA and TOA was different by month and time of the day, respectively. All statistical analyses were two-sided. p-value<0.05 was considered to be statistically significant.

## RESULTS

**Figure 2:**
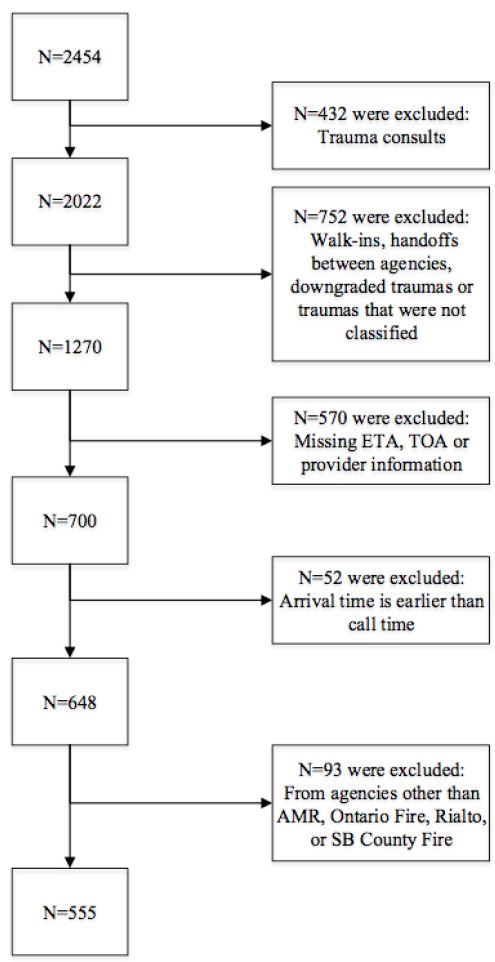
Patient inclusion criteria flow chart. *ETA*, estimated time of arrival; *TOA,* time of arrival; *AMR* American Medical Response; *SB County Fire*, San Bernardino County Fire.

A total of 2,454 patients classified as traumas were identified in the Surgery Department Trauma Registry between January 1, 2013, and December 31, 2013. After exclusion of trauma consults (n=432), walk-ins, handoffs between agencies, downgraded trauma or traumas that were not classified (n=752), traumas with missing ETA, TOA or provider information (n=570), traumas where the arrival time was noted as earlier then the call time (n=52), traumas transported by agencies other than AMR, Ontario Fire, Rialto Fire, or San Bernardino County Fire (n=93), we included a sample size of 555 trauma alerts and activations in the final analysis. (See Figure 2 for patient flow chart.)

**Figure 3:**
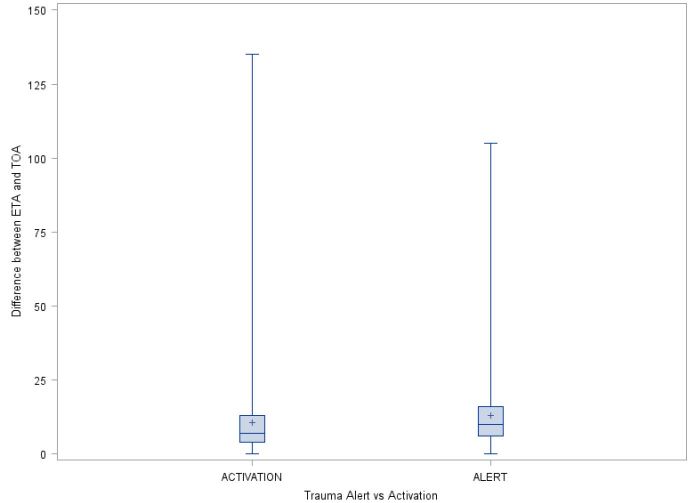
The boxplot of difference between the estimated time of arrival (ETA) and time of arrival (TOA) by trauma alerts or activations. *p<0.0001 for the effect of alert vs. activation on the difference of median between ETA and TOA.

When combining all transports by the included EMS agencies, the median of the ETA was 10 minutes, whereas the median of the actual TOA was 22 minutes (Table). There is a statistically significant difference between median of the estimated and actual time of arrival (median of difference=9 minutes, p<0.0001). For each EMS agency, there are statistically significant differences between the median of the estimated and actual time of arrival (p<0.0001 for all four EMS agencies). San Bernardino County Fire had the largest difference of 11 minutes and Rialto Fire had the smallest difference of six minutes. Additionally, for each EMS agency and for all four agencies combined, transports classified as trauma alerts had a larger or equal median of the estimated and actual time of arrival than those transports classified as trauma activations. Specifically, for all four EMS agencies combined, the medians of difference between ETA and TOA was 10 and seven minutes for alerts and activations, respectively (p<0.0001, Figure 3).

**Figure 4:**
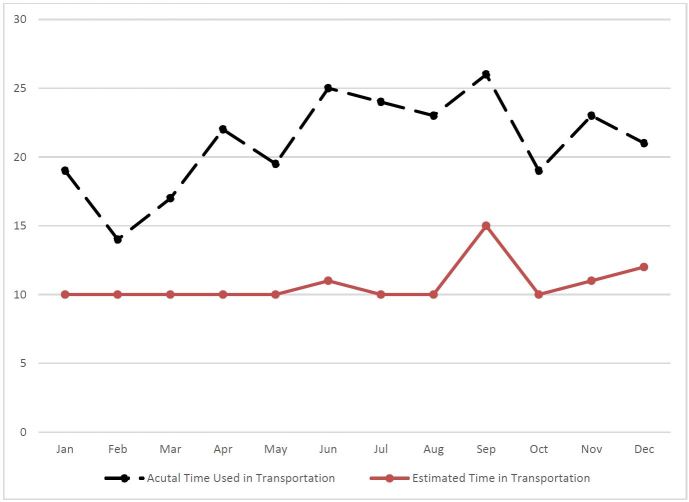
The median of actual and estimated transport time by month.

**Figure 5:**
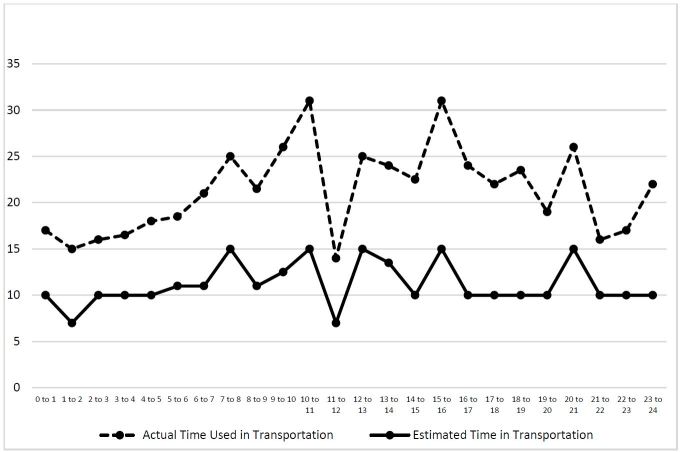
The median of actual and estimated transportation time by time of the day.

We conducted two more analyses to identify the median ETA and TOA by month and time of the day, respectively (Figures 4 and 5). Both month and time of the day were associated with the difference between the median of the estimated and actual arrival time (p=0.0082 and p=0.0005 for month and time of the day, respectively). The difference between these two medians peaked in June (the median difference was 12 minutes), and was smallest in February (the median difference was four minutes). Additionally, the difference between these two medians peaked at 10 to 11AM (the median difference was 16 minutes, followed by 7 to 8AM (the median difference was 14 minutes).

## DISCUSSION

For alert and activation classified traumas, the findings of this study show that the predicted travel time by EMS personnel from the scene to the hospital is often significantly underestimated. In the majority of transports, providers arrived to the hospital after their estimated arrival time. This results in early, and often prema ture, trauma system activation. Across nearly 2,500 trauma alerts and activations transported to ARMC in 2013, an average discrepancy of nine minutes between the estimated arrival time and actual arrival time for each trauma case has the potential to interrupt the flow of ED patient care and create significant ripple effects throughout daily hospital operations. An average of seven trauma alerts or activations per day would lead to one hour of “wait time” per day by the trauma team and around 30 hours per month. With at least eight personnel, including ED and surgery department staff, arriving for each trauma alert or activation (Figure 1), this amounts to 240 hours of total “wait time” per month and over 2,800 hours per year.

Further data analysis noted a difference in the discrepancy between the estimated arrival time and actual arrival time when comparing trauma alerts and activations. EMS personnel estimated their arrival time with a greater degree of accuracy when transporting trauma activations. To our knowledge, the impact of trauma classification on the accuracy of the estimated arrival time has not been assessed in previous studies. Factors impacting this association may be assessed in future investigations.

One could contend that there are positive aspects to an underestimation of transport time by EMS personnel leading to an early activation of a trauma team and mobilization of hospital resources. Early arrival of a trauma team to the ED prior to a trauma patient’s arrival provides time to assign roles, prepare equipment for resuscitation and set up radiological equipment. Early activation can also facilitate logistical preparation for an arriving patient. Previous investigations have shown that timely trauma system activation improves the trauma team performance as measured by time to chest radiograph.^16^ However, it has further been determined that proactive trauma team activation and subsequent early trauma team arrival and mobilization of resources has no effect on ED length of stay and mortality in most patients.^16^,^17^ Yet despite no noted increase in ED length of stay, early trauma team activation may be important in select cases such as when multiple trauma patients arrive simultaneously or for stroke and myocardial infarction cases when door-to-needle time could potentially be shortened.

Nevertheless, potentially negative factors must be weighted when assessing an underestimation of transport time and early trauma system activation. Previous studies have demonstrated that emergency physicians would alter their prehospital-directed medical management of an incoming patient 8.5% of the time if a more accurate ETA were given.^18^ Further, the trauma systems at ARMC are activated 15 minutes prior to patient arrival when possible and the trauma team (Figure 1) is expected to arrive in the ED within 1–3 minutes. An additional nine minutes of “wait time,” often greater than 6–8 times per day, can repeatedly divert clinicians and staff away from patient care, as well as interrupt or delay surgeries, reducing work flow not only in the ED but throughout the hospital. It has been shown that trauma system activation increases the ED length of stay by an average of 16 minutes for other patients requiring admission who arrived within three hours before or after trauma patient arrival.^11^ Additionally, hospital imaging services are often placed on standby for a trauma patient’s potential need.^11^ A computed tomography scanner frequently placed on hold for nine minutes significantly reduces the number of patients who can receive timely care. In combining the effect of delayed physician evaluations with the priority reservation of imaging resources, operating rooms and laboratory services for trauma patients, these factors have the potential to further increase the length of time to discharge or admission. Previous studies have shown that ED crowding can influence ED and inpatient outcomes, including patient mortality.^19^–^21^ ED crowding has also been associated with an increased cost of inpatient care.^20^ Though a discrepancy in ambulance arrival time is not the only factor leading to ED crowding, it is undoubtedly a contributing factor. Understanding discrepancies in arrival times is one step toward a solution to this multifactorial, systemic issue.

At present, there appears to be no standardized aids or protocols for EMS personnel or ED staff to reliably anticipate the travel time and estimated arrival time of trauma patients. Though radio or telephone contact presents as an initial means of communication, there is the potential for EMS crews to be preoccupied with resuscitation efforts and unable to provide timely communication. Additionally, the current system forces EMS personnel to estimate delays due to traffic conditions and weather fluctuations. This assumes that crews have sufficient and up-to-date information concerning potential sources of delay.

As technology advances and becomes readily accessible, implementation of real-time global positioning systems (GPS) available to the ED staff to follow EMS vehicles presents as a possible solution to provide a consistent and accurate arrival time. GPS is already used in prehospital care for strategic deployment of ambulances, as well as in the development of ambulance deployment protocols and placement of helipads for air medical services.^22^,^23^ Further, the effectiveness of GPS tracking to predict ambulance arrival time to a trauma center was demonstrated through the development of a web-based application that integrates GPS tracking of ambulances and Google Maps. This model took into account factors such as local traffic, time of day and use of lights and sirens. Through a retrospective analysis of nearly 50,000 patient transports, investigators were able to use this model to predict arrival time within five minutes 72.8% of the time.^24^ A further retrospective study validated the use of Google Maps and other methods for route-based transport time estimation, noting the use of Google Maps as moderately accurate with a mean absolute error of 3.5 minutes for transport time estimation.^25^ Based on these investigations, it appears resourceful and plausible to implement a similar system to diminish findings in this study regarding the difference between ETA and TOA between EMS crews and ED staff in San Bernardino County, CA.^26^

At present, a majority of the fire and EMS providers in San Bernardino County optimize vehicle deployment through a computer-aided dispatch (CAD) system in conjunction with satellite tracking via GPS and automatic vehicle locators (AVL). It is conceivable that these data could be shared with ED staff at ARMC. Sharing of this data would not violate Health Insurance Portability and Accountability Act regulations, as ambulance arrival time is relevant to trauma patient care. A challenge would be to create a system that is compatible with the current fire and EMS provider infrastructure. A final obstacle is the financial expense associated with the development of this new system. In considering these logistical and financial factors, initial implementation of GPS tracking available to ED staff could be undertaken and studied in a single agency with a high volume of transports in order to assess the accuracy and benefit of arrival time prediction with GPS tracking.

## LIMITATIONS

This study was subjected to several limitations. One limitation was a lack of complete information (a time of initial contact, ETA and name of the transporting provider) associated with each patient transport. We excluded 570 patients from the four included agencies due to missing data regarding the calculation of ETA and TOA. These excluded patients were similar to those included in the final analysis with respect to age, gender, mechanism of injury and time of day. As such, we believe that the included sample is a random sample of patients transported by EMS agencies and is representative of the actual situation based on the distribution of the data.

Furthermore, this study used a single hospital trauma care registry as the primary data source that relies on the notation of time entries on paper by ED staff during initial patient management. This may impact the generalizability of the findings in this study. Additional studies are warranted to validate these results in other EMS systems and explore possible solutions for more effective travel time prediction. Further, data are later entered manually into an electronic database - the Surgery Department Trauma Registry. In terms of data quality, manually recorded data has the potential for human error. Previous studies alluded to the fact that manually recorded data are subject to the human propensity to smoothen data.^16^,^27^ For future studies, it may be beneficial for the trauma registry to move toward automatic capturing of time data.

One important aspect of the analysis to consider, also highlighted by previous studies, is the interpretation of ETA communicated by EMS as a time interval.^18^ For example, an ETA given as 10 to 15 minutes can be taken as either of the two extremes or the midpoint. In this study, we chose to use the midpoint for consistent data analysis. As a result, more values will have a positive or negative “difference in time” despite falling within the given ETA interval. However, we believe that similar results would have been reached regardless of the ETA data parameter.

A parameter that we were unable to assess was the impact of the use of lights and sirens by EMS personnel on the accuracy of predicted travel time. EMS crews are not required to use lights and sirens when transporting trauma alerts and activations in San Bernardino County. However, previous studies have shown that the effect of lights and sirens does not have a significant impact on transport time for most transports. Lights and sirens have been shown to affect transport time in longer transports.^28^,^29^ The impact of lights and sirens on predicted travel time could be assessed in future studies.

## CONCLUSION

The findings demonstrated that EMS personnel consistently underestimated travel time leading to a discrepancy in their estimated arrival time and actual arrival time. This resulted in the premature activation of trauma systems in the majority of trauma alert and activations transported to ARMC. In turn, hospital personnel and trauma teams waited longer for trauma patient arrival, delaying the care of other patients and diverting hospital resources for more time than necessary.

Overall, this study calls attention to a systemic concern surrounding inaccurate ambulance arrival times. It is clear that we must determine a way to accurately and consistently predict patient arrival, regardless of whether patients frequently arrive before or after their predicted arrival time to any hospital. With advancing technology, GPS represents an immediately plausible, accurate and reproducible solution. Reducing discrepancies in ambulance arrival time is one factor that will lead us toward tackling the multifactorial causes of crowding and increased wait times in emergency departments across the United States.

## Figures and Tables

**Table:** Median estimated time of arrival (ETA) in comparison to median time of arrival (TOA) for all included emergency medical service (EMS) agencies: American Medical Response (AMR), Ontario Fire, Rialto Fire and San Bernardino County Fire (SB County Fire).

	Median ETA (min)			Median TOA (min)			Median difference			
		
EMS agency	Alert	Activation	Combined	Alert	Activation	Combined	Alert	Activation	Combined	p-value
AMR	10	10	10	23	18	21	9	7	9	<0.0001
Ontario Fire	20	15	20	31.5	22	28	10	7	9	<0.0001
Rialto Fire	5	5	5	12	12	12	8	6	6	<0.0001
SB city Fire	15	10	11	30	19.5	25	14	8	11	<0.0001
All 4 EMS agencies combined	12	10	10	24	18	22	10	7	9	<0.0001

* p-value was calculated to test whether the combined median difference was significantly different from zero. In other words, whether the median of estimated time of arrival and time of arrival are the same for each agency separately and for all four EMS agencies combined.

** Median difference is calculated as the median of the difference between ETA and TOA (using ETA-TOA). We calcualted ETA-TOA, then we calculated the median of these differences.

## References

[1] (2014). Trauma Statistics.

[2] Injury Prevention and Control: Data and Statistics (WISQARS).

[3] Uleberg O, Vinjevoll O, Eriksson U (2007). Overtriage in trauma - what are the causes?. Acta Anaesthesiol Scand.

[4] Jenkins P, Kehoe A, Smith J (2013). Is a two-tier trauma team activation system the most effective way to manage trauma in the UK?. J Trauma.

[5] Mann N, Mullins R, MacKenzie E (1999). Systematic review of published evidence regarding trauma system effectiveness. J Trauma.

[6] Celso B, Tepas J, Langland-Orban B (2006). A systematic review and meta-analysis comparing outcome of severely injured patients treated in trauma centres following the establishment of trauma systems. J Trauma.

[7] MacKenzie E, Rivara F, Jurkovich G (2006). A national evaluation of the effect of trauma-centre care on mortality. N Engl J Med.

[8] Lendrum R, Lockey D (2013). Trauma system development. Anaesthesia.

[9] Rainer T, Cheung N, Yeung J (2007). Do trauma teams make a difference?. Resuscitation.

[10] Keating J, Anderson I, Egan G (2012). Trauma Care in Scotland.

[11] Smith D, Chapital A, Uperesa B (2011). Trauma activations and their effect on non-trauma patients. J Emerg Med.

[12] Jurkovich G, Campbell D, Padrta J (1987). Paramedic perception of elapsed field time. J Trauma.

[13] Lee C, Walters E, Borger R (2016). The San Bernardino, California, Terror Attack: Two Emergency Departments’ Response. West J Emerg Med.

[14] (2014). San Bernardino County 2014 Community Indicators Report.

[15] (2015). State and County Quick Facts - San Bernardino County, California.

[16] Lillebo B, Seim A, Vinjevoll O (2012). What is optimal timing for trauma team alerts? A retrospective observational study of alert timing effects on the initial management of trauma patients. J Multidiscip Healthc.

[17] Liberman M, Mulder D, Jurkovich G (2005). The association between trauma system and trauma center components and outcome in a mature regionalized trauma system. Surgery.

[18] Propp D, Rosenberg C (1991). A comparison of prehospital estimated time of arrival and actual time of arrival to an emergency department. Am J Emerg Med.

[19] Bernstein SL, Aronsky D, Duseja R (2009). The effect of emergency department crowding on clinically oriented outcomes. Acad Emerg Med.

[20] Sun BC, Hsia RY, Weiss RE (2013). Effect of emergency department crowding on outcomes of admitted patients. Ann Emerg Med.

[21] Singer AJ, Thode HC, Viccellio P (2011). The Association Between Length of Emergency Department Boarding and Mortality. Acad Emerg Med.

[22] Ong M, Chiam T, Ng F (2010). Reducing ambulance response times using geospatial-time analysis of ambulance deployment. Acad Emerg Med.

[23] Foo C, Ahghari M, MacDonald R (2010). Use of geographic information systems to determine new helipad locations and improve timely response while mitigating risk of helicopter emergency medical services operations. Prehosp Emerg Care.

[24] Fleischman R, Lundquist M, Jui J (2013). Predicting Ambulance Time of Arrival to the Emergency Department Using Global Positioning System and Google Maps. Prehosp Emerg Care.

[25] Wallace DJ, Kahn JM, Angus DC (2014). Accuracy of prehospital transport time estimation. Acad Emerg Med.

[26] Cone D, Landman A (2014). New Tools for Estimating the EMS Transport Interval: Implications for Policy and Patient Care. Acad Emerg Med.

[27] Schalkwyk Jv, Lowes D, Frampton C (2011). Does mannual anaesthetic record capture remove clinically important data?. Br J Anaesth.

[28] Hunt RC, Brown LH, Cabinum ES (1995). Is ambulance transport time with lights and siren faster than that without?. Ann Emerg Med.

[29] O’Brien DJ, Price TG, Adams P (1999). The effectiveness of lights and siren use during ambulance transport by paramedics. Prehosp Emerg Care.

